# Evidence certainty in neonatology—a meta-epidemiological analysis of Cochrane reviews

**DOI:** 10.1007/s00431-025-06023-w

**Published:** 2025-02-11

**Authors:** Tuomas Varrio, Daniele De Luca, Ilari Kuitunen

**Affiliations:** 1 Kuopio Pediatric Research Unit, University of Eastern Finland, Kuopio, Finland; 2 Division of Pediatrics and Neonatal Critical Care, “A.Beclere” Hospital, APHP-Paris Saclay University, Paris, France; 3 Physiopathology and Therapeutic Innovation Unit-INSERM U999, Paris Saclay University, Paris, France; 4 Department of Pediatrics and Neonatology, Kuopio University Hospital, Puijonlaaksontie 2, 142072 Kuopio, Finland

**Keywords:** Meta-epidemiology, Neonatology, Evidence-based medicine

## Abstract

We hypothesized that certainty of the available evidence is relatively low in neonatology. Thus, we designed a meta-epidemiological review to examine what is the certainty of evidence in the latest Cochrane neonatal reviews and investigate if the number of trials and enrolled patients is associated with the certainty of evidence. We searched Cochrane neonatal reviews published between January 2022 and May 2024. We included all reviews on interventions concerning neonates that had at least one meta-analysis performed with GRADE-rated evidence certainty. From those reviews, we extracted the presented certainty of evidence and analyzed its association with the number of trials and participants by ANOVA. We screened 55 Cochrane reviews and included 49 of them. In these 49 reviews, there were 443 reported outcomes with graded certainty of evidence. The certainty was reported to be high in 8 (1.8%), moderate in 89 (20.2%), low in 195 (44.0%), and very low in 151 (34%) of the outcomes. Reviews reporting outcomes with higher certainty of evidence had significantly more trials and patients (approximately 3 and 1.5 times more, respectively) than those with only low certainty of evidence.

*Conclusion*: In the past 2 years, Cochrane neonatal reviews have generally had low or very low certainty of evidence for most outcomes. Only 2% of the reviewed outcomes had high certainty. The number of included patients and trials significantly affected the certainty. These findings highlight the continuous need for better quality and larger trials.

**Table:** 

**What Is Known:** *• Neonatology is among the largest specialities and the evidence certainties of interventions have been varying.* *• Neonatal patients and studies need to consider the uniqueness of the patients and the acute situations in the study designs.* **What Is New:** *• The included 49 reviews consisted of 443 outcomes and of these only 1.8% were classified as high certainty of evidence.* *• Higher evidence certainties were associated with higher number of included trials and participants.*

**Supplementary Information:**

The online version contains supplementary material available at 10.1007/s00431-025-06023-w.

## Introduction

Clinical care moved away from traditional intuition-based approaches towards evidence-based medicine (EBM) [1]. EBM aspires to provide the best possible care for patients, supported by high-quality evidence. Nonetheless, high-quality evidence is rather an unclear term, and thus, objective measurements of its certainty have been introduced. The most used framework to address evidence certainty is the Grading of Recommendations Assessment, Development and Evaluation (GRADE). GRADE is crucial in translating scientific evidence into medical recommendations and is also a valuable tool to help clinicians better understand the gathered evidence [2].

High-quality randomized controlled trials (RCTs) represent the basis of the foundation of evidence [3]. Since the beginning of the twenty-first century, the publication rate of high-quality research concerning neonates has been declining [4]. Many factors contribute to this, but the most significant are the rising standards to define the quality of clinical research, which mainly evolved in adult medicine and are not easy to be reproduced in neonatology. In fact, the high certainty of evidence consists typically of several high quality and large randomized controlled trials, whose results are in consensus with each other. Nonetheless, this is uncommon in neonatal trials due to varying co-interventions and definitions for multifaceted and multifactorial outcomes (e.g., bronchopulmonary dysplasia) [2], This variation makes it difficult to combine results and estimate the effectiveness of interventions and the certainty of the evidence [2]. Being born extremely prematurely usually means that the newborn is in a critical condition and has many medical problems occurring simultaneously and influencing each other [5]. Therefore, it is challenging for a single intervention to significantly impact on an outcome, making it unfair to deem these interventions unsuitable in neonatology [5]. Thus, we hypothesize that certainty of the available evidence is relatively low, and we designed a meta-epidemiological review to examine what has been the evidence certainty in the latest Cochrane neonatal reviews and investigate if the number of trials and enrolled patients is associated with the certainty of evidence.

## Methods

### Protocol

We performed a systematic meta-epidemiological review, whose protocol was registered in Open Science Framework, and it is available from https://osf.io/7k6s8/. The protocol was agreed before commencing the search and included search criteria, analysis plan, and full description of methods.

### Search process and screening

For this work, we searched Cochrane neonatal reviews published between January 2022 and May 2024 from the Cochrane review register. We decided to focus on the past 2 years to provide the most recent view of neonatal evidence. Furthermore, we hypothesized that these reviews would be similar in terms of methodology, considering that guidance on risk of bias was updated in 2019, and GRADE guidance is continuously updated. The search results were then uploaded to Covidence software (Veritas Healthcare, Melbourne, Australia) for abstract screening. Two authors (TV and IK) performed the screening process independently, and disagreements, if any, were solved by reaching a mutual consensus or consulting the third author.

### Inclusion and exclusion criteria

We included all Cochrane reviews on interventions that targeted neonates and had at least one meta-analysis performed for which the evidence certainty was rated according to GRADE criteria. If the review remained qualitative and did not perform any statistical pooling of the results, it was excluded.

### Data extraction and treatment

Data from the reviews were extracted to a pre-designed Excel spreadsheet. First, 20% of the reviews were extracted independently by two authors (TV and IK) to pilot and test the extraction process, and as there were no conflicts, one author (TV) extracted the remaining 80% of the review data. The following information was extracted from each included review and for each outcome: year of publication, intervention, control, patient population, setting, number of studies, number of participants, effect estimates, and evidence certainty rating as declared by the meta-analysis according to GRADE classification [6]. This information was extracted from the presented summary of findings tables.

We classified the patient populations analyzed by each Cochrane meta-analysis in three groups as follows: (1) preterm neonates (gestational age less than 37^+0^ weeks), (2) term neonates (gestational age 37^+0^ weeks or more), (3) neonates of any gestational age. Furthermore, the meta-analyzed interventions were classified as ventilation, nutrition, medication, and others. Finally, the outcomes were classified as “subjective” or “objective” for the outcome assessor. Objective outcomes were, for example, laboratory parameters and clinical measurements which are measured by standardized methods. Death, intraventricular hemorrhage, and bronchopulmonary dysplasia were also considered as objective clinical outcomes. On the contrary, outcomes that may be possibly affected by the knowledge of the received intervention were considered subjective (e.g., duration of invasive ventilation, need for reintubation, time to discharge, pain). For full transparency, all the extracted data is available in the online supplementary file 1.

### Statistics

For categorized variables, we presented absolute numbers with proportions (%). We used cross tabulation and Chi-squared or Fisher test, as appropriate, to examine differences in categorical outcomes between groups. We presented the findings for different interventions and outcomes in a traffic-light plot where green indicates high, yellow indicates moderate, orange indicates low, and red indicates very low certainty. The mean number of studies and patients per each evidence certainty categories were analyzed with ANOVA followed by Bonferroni post hoc test. A sensitivity analysis with Kruskal–Wallis test was also made. Statistical analyses were made by using SPSS 29.0 (IBM, Chicago-IL, USA), and *p* value less than 0.05 was considered statistically significant. We have reported the findings of this review according to the meta-epidemiological extension of the preferred reporting items in systematic reviews and meta-analysis (PRISMA) guideline [7].

## Results

**Fig. 1:**
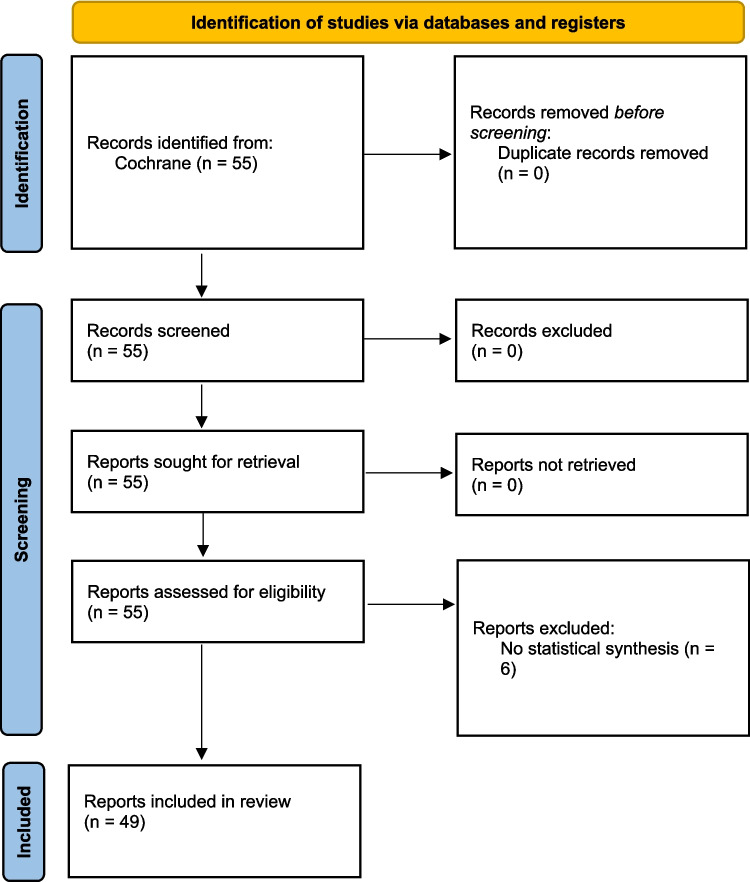
PRISMA flowchart of the study selection process

We screened 55 Cochrane reviews and included 49 of these for our analysis; six articles were excluded due to lack of quantitative meta-analysis in the review (Fig. 1). The included 49 reviews reported a total of 443 outcomes whose certainty of evidence was evaluated. Of the included articles, 14 reviews with 115 outcomes focused on feeding interventions, 8 reviews with 75 outcomes on ventilation, 17 reviews with 180 outcomes on medications, and 10 reviews with 73 outcomes were classified as miscellaneous.

**Fig. 2:**
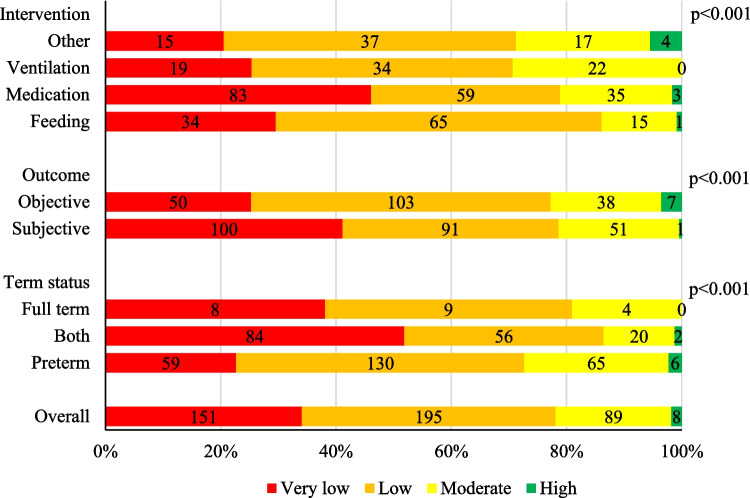
Traffic-light plot of the evidence certainties. Evidence certainties are classified by type of intervention, outcome subjectivity, and patient maturity; differences are tested by chi-squared/Fisher test (more details in the main text). Overall certainties are also depicted. Absolute numbers presented in the graph and the scale is proportional to 100% for colors

Overall, the certainty of evidence was reported to be high in 8 (1.8%), moderate in 89 (20.2%), low in 195 (44.0%), and very low in 151 (34.0%) of the outcomes (Fig. 2). There were significant differences in certainty of evidence among reviews focusing on different interventions (*p* < 0.001). For instance, the highest proportion of very low certainty was in the medication reviews, and the highest proportion of low certainty was in feeding reviews; ventilation and other interventions had rather similar certainty (Fig. 2). There was no high certainty of evidence outcomes in ventilation reviews. Reviews reporting subjective outcomes had a significantly greater proportion of very low certainty (100 out of 243, i.e., 41.2%) than those with objective outcomes (50 out of 198, i.e., 25.3%, *p* < 0.001), and, finally, certainty was best in the reviews focusing solely on preterm neonates (Fig. 2).

**Fig. 3:**
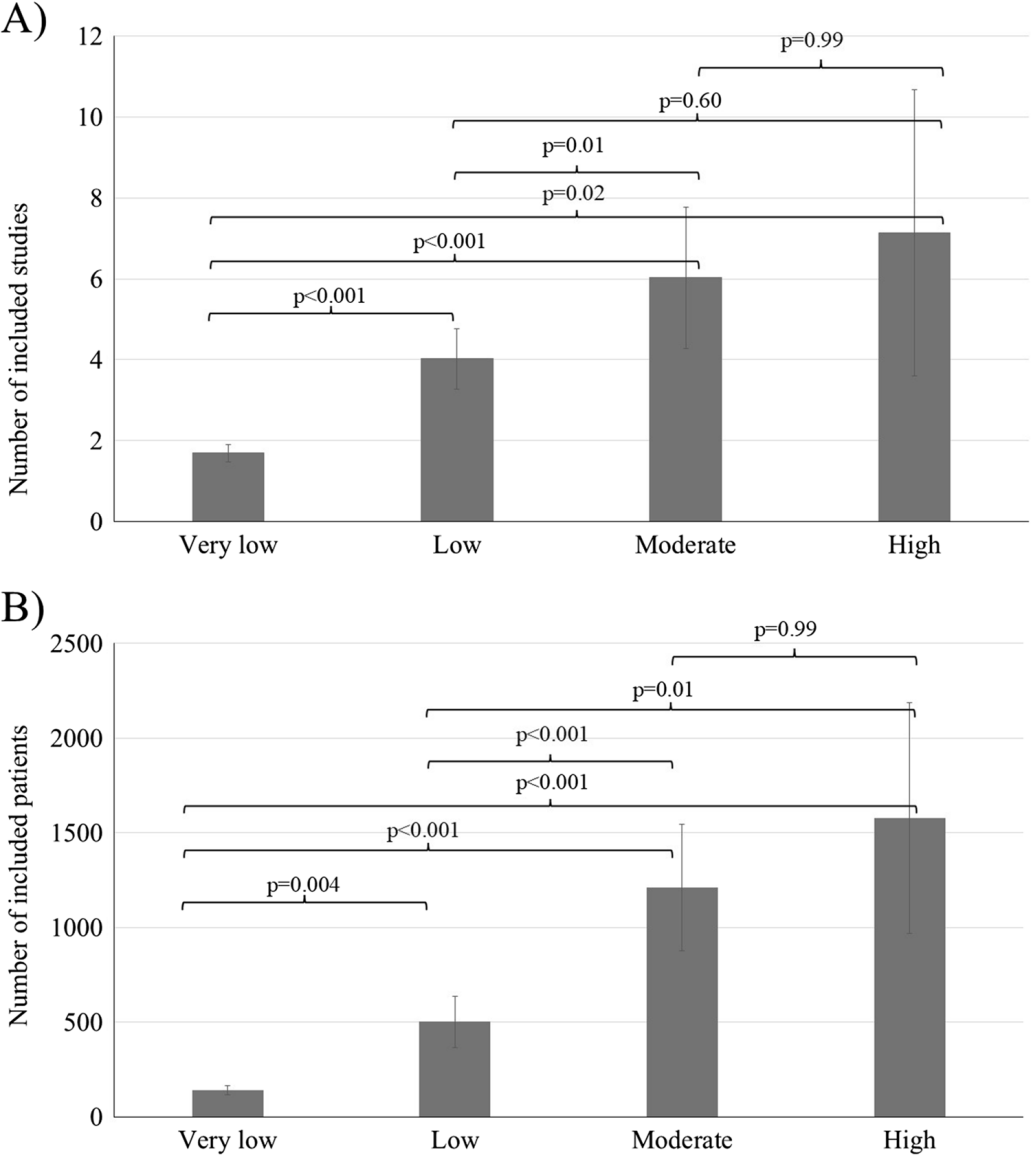
Average number of trials (**A**) and participants (**B**) among outcomes with a reported certainty of evidence. Columns and T-bar represent means and 95% confidence intervals (CIs), respectively. Horizontal lines represent significant post hoc comparisons. Data are analyzed with ANOVA and Bonferroni post hoc test

Reviews with at least one outcome with high certainty of evidence included significantly more trials and enrolled patients compared to those with low certainty (*p* < 0.001 for both the number of studies and patients; Fig. 3A, B, respectively). Results of post hoc analyses are shown in Fig. 3: numbers of studies and patients were significantly different between outcomes of any certainty of evidence with the only exception of moderate *vs* high certainty comparisons. A sensitivity analysis with Kruskal–Wallis test showed similarly *p* < 0.001 for both the number of studies and patients. Specifically, reviews with at least one outcome with high certainty had approximately 3 and 1.5 times more studies or patients than those with very low or low certainty, respectively.

Outcomes with high certainty of evidence were eight, and their details are presented in Table 1. The interventions which had high certainty for at least one outcome were early developmental intervention programs, lumbar puncture position, oral dextrose gel, methylxanthine for apnea prevention, musical/vocal interventions for preterm neonates, and indomethacin and ibuprofen in PDA patients. Interestingly, only four (50%) of the high-certainty evidence outcomes were based on a pharmacological intervention. Furthermore, four reviews reported null findings, as they reported no clear difference between the intervention and control groups.

**Table 1:** Outcomes with high certainty of evidence in neonatal Cochrane reviews published in 2022–2024

Review	Patients	Intervention	Control	Outcome	*N* of studies	*N* of participants	Effect estimate
Early developmental intervention programs provided post hospital discharge to prevent motor and cognitive impairment in preterm infants	Preterm infants	Early developmental intervention programs	Standard follow‐up	Cognitive outcome at preschool age—IQ (Bayley, Stanford‐Binet, McCarthy, WPPSI‐III)	9	1524	SMD 0.39 higher (0.29 higher to 0.5 higher)
Positioning for lumbar puncture in newborn infants	Lumbar puncture in newborn infants	Lateral position	Sitting position	Time to perform the lumbar puncture	2	1102	MD 2.00, 95% CI − 4.98 to 8.98
Oral dextrose gel to prevent hypoglycemia in at‐risk neonates	Newborn infants at risk of neonatal hypoglycemia	Dextrose gel	placebo gel	Hypoglycaemia (investigator‐defined)	2	2548	RR 0.87 (0.79 to 0.95)
Methylxanthine for the prevention and treatment of apnea in preterm infants	Preterm infants at risk of or with apnea; preterm infants undergoing extubation	Methylxanthine	Placebo or control	Supplemental oxygen at 36 weeks’ postmenstrual age	4	2142	RR 0.77 (0.69 to 0.85)
Musical and vocal interventions to improve neurodevelopmental outcomes for preterm infants	Preterm infants and their parents	Music/vocal interventions	Standard care	Oxygen saturation during intervention	10	958	MD 0.13 higher (0.33 lower to 0.59 higher)
Musical and vocal interventions to improve neurodevelopmental outcomes for preterm infants	Preterm infants and their parents	Music/vocal interventions	Standard care	Heart rate postintervention assessed up to 30 min	9	903	MD 3.8 lower (5.05 lower to 2.55 lower)
Prophylactic cyclo‐oxygenase inhibitor drugs for the prevention of morbidity and mortality in preterm infants: a network meta‐analysis	Preterm or low birth–weight infants without a prior clinical or echocardiographic diagnosis of PDA	Indomethacin	Placebo	Necrotizing enterocolitis	14	2543	RR 0.76 (0.35 to 1.2)
Prophylactic cyclo‐oxygenase inhibitor drugs for the prevention of morbidity and mortality in preterm infants: a network meta‐analysis	Preterm or low birth–weight infants without a prior clinical or echocardiographic diagnosis of PDA	Ibuprofen	Placebo	Necrotizing enterocolitis	7	905	RR 0.73 (0.31 to 1.4)

## Discussion

In general, we found that most outcomes in the Cochrane neonatal reviews published in the last 2 years have low or very low certainty of evidence. Only about 2% of the outcomes had high certainty, and around 20% had moderate certainty. These percentages varied slightly across different categories, such as type of intervention, but the overall proportions of certainty were almost identical within these categories. We also found a significant association between certainty of evidence and the number of trials and enrolled patients. However, our main finding was the worrisome and striking low number of reviews with high certainty for different interventions and outcomes. For example, none of the studies on ventilation interventions had high certainty of evidence.

There are many possible explanations for this result. First, there is a high variance in the number of trials comparing different non-invasive and invasive ventilation modalities. For example, the use of non-invasive neurally adjusted ventilation and nasal high frequency oscillatory ventilation was studied only in 5 small trials and approximately 10 randomized studies, respectively [8–11]. Second, the understanding of the outcome assessments subjectivity plays a key part, as ventilation strategies are hard to be blinded to attending clinicians. A recent meta-epidemiological review on this matter found a worrying rate of incorrectly performed risk of bias assessment leading to downgrading of certainty [12], and the proportion of incorrect assessments was similar between Cochrane and non-Cochrane reviews. Nonetheless, the recent NASONE trial showed that it is possible to compare complex ventilation strategies for a long time with an assessor-blinded design and reduce possible bias [9]. More and above this, it is important to remind that ventilation cannot be considered as a single intervention, like a pharmaceutical therapy. Ventilation is a complex technique delivered on various modes and, even within the same mode, with several possible combinations of ventilatory parameters and thus several therapeutic strategies. This is even more complex for non-invasive ventilation where the use of different interfaces plays a significant role in ventilation efficacy, patient-ventilator interaction, and comfort [13, 14]. The lack of detailed standardization of the ventilatory intervention can significantly downgrade the quality of results, and only recent trials have implemented this concept [9, 15]. Finally, a ventilatory strategy may be applied to neonates with completely different lung mechanics and pathophysiology (e.g., respiratory distress syndrome or bronchopulmonary dysplasia or neonatal acute respiratory distress syndrome): this also plays a role in downgrading the quality and demands a close pathophysiology phenotyping to enroll homogeneous populations [16, 17]. This is the basis for a personalized neonatal respiratory care and currently represent an important need [18].

The number of trials and enrolled patients was associated with the certainty of evidence. In other words, outcomes that were more widely studied had higher certainty. This is consistent with previously published meta-epidemiological reports and is worrisome. In fact, as previous reports date to 2006 and 2013, the situation has not significantly improved over time [19, 20].

As previously reported, the majority of the studies and reviews focus on preterm neonates. In fact, critically ill preterm neonates are more numerous than term infants, and the two age groups present with very different diseases and comorbidities. Thus, not only preterm neonates are more commonly admitted to NICUs than term babies, but these latter present with more complex and rare life-threatening conditions such as, for instance, meconium aspiration [21, 22] that are more difficult to study in randomized trials. The association between the certainty of evidence and the number of trials and enrolled patients was expected since, according to the GRADE classification, an intervention needs a sample size large enough to produce precise results [23]. One of the domains in the GRADE assessment is the imprecision of the outcome estimate. As imprecision is mostly based on the interpretation of the effect estimates, whose confidence intervals are narrower for more common event and larger populations. It is also interesting that four of the eight high certainty outcomes reported the “so called” null findings, where the analyzed intervention did not show evidence of a difference compared to control intervention.

The classification of the certainty of evidence may change if reviews are updated, and the outcome estimates usually become more precise [24]. It is more common for very low evidence to be reclassified into higher categories, than for high evidence outcome to be downgraded [12, 25]. Continuous research efforts to improve the quality of neonatal care are needed. Traditionally, this means designing more and larger randomized trials. However, meta-epidemiological reviews of existing literature are important, as these hold potential to spare resources from unnecessary trials.

### Strengths and limitations

The main strength of our work was the full transparency and respect of best review practices without any protocol violations. The main limitation comes from the fact that we classified the review interventions and outcomes after data extraction and classified the outcomes as subjective and objective based on our own experience and opinion. To reduce this and promote transparency, we have provided the whole data as a supplementary file. Furthermore, a further limitation was that we had a protocol deviation, as we initially planned to use dual data extraction but performed the data extraction by individual author after 20% of the reports were extracted. This decision was based on the fact that there were no issues with the extraction as it was made directly from the summary of findings tables as those were. Another limitation may be that we decided to focus solely on Cochrane reviews and on a relatively narrow amount of time (i.e., 2022–2024). We decided so because (1) Cochrane reviews are conducted rigorously using the same formats and protocols, and (2) we wanted to have a picture of the really recent situation. In fact, we considered that the risk of bias tool was revised in 2019 and implemented into use only in 2020; this means that the protocols for the reviews published in 2022 have been written mostly in 2020–2021. However, it could be that the results here are influenced by the short timeline, and a longer study period could have altered the results in either direction. Thus, a future study should investigate if results presented here may be different on a wider time window or comparing Cochrane and non-Cochrane meta-analyses. A clear limitation of our results was the lack of detailed extraction on the GRADE domain assessments and the reasons for downgrading the evidence certainty. Although these were provided in the Cochrane reviews, we did not extract this information, and this will be the object of future meta-epidemiological studies. Thus, future research should analyze the GRADE assessments more in detail and clarify the reasons of downgrading the certainty and whether these decisions have been correctly taken.

## Conclusion

Only 2% of the outcomes had high certainty of evidence in the Cochrane neonatal reviews published in the last 2 years. The certainty was significantly associated with the number of included trials and participants. Neonatal trials and reviews still need more and larger studies to improve the overall quality of the evidence.

## Supplementary Information

Below is the link to the electronic supplementary material.

Supplementary file1 (XLSX 73 KB)

## Data Availability

All extracted data available as a supplementary file.

## References

[1] Saugstad OD, Kirpalani H (2023) Searching for evidence in neonatology. Acta Paediatr 112(8):1648–1652 37151193 10.1111/apa.16815

[2] Soll RF, Ovelman C, McGuire W (2020) The future of Cochrane Neonatal. Early Hum Dev 150:105191. 10.1016/j.earlhumdev.2020.105191 33036834 10.1016/j.earlhumdev.2020.105191

[3] Schumacher RE (2011) Myth: Neonatology is evidence-based. Semin Fetal Neonatal Med 16(5):288–292 21636335 10.1016/j.siny.2011.04.005

[4] Willhelm C, Girisch W, Gortner L, Meyer S (2012) Evidence-based medicine and Cochrane reviews in neonatology: Quo vadis? Acta Paediatr 101(4):352–353 22150748 10.1111/j.1651-2227.2011.02559.x

[5] Meyer S, Gottschling S, Gortner L (2008) Evidence-based medicine in neonatology: time to re-think. Eur J Pediatr 167(9):1089–1089 18301919 10.1007/s00431-008-0692-3

[6] Guyatt GH, Oxman AD, Vist GE, Kunz R, Falck-Ytter Y, Alonso-Coello PYM et al (2008) GRADE: an emerging consensus on rating quality of evidence and strength of recommendations. BMJ 336(7650):924–6 18436948 10.1136/bmj.39489.470347.AD PMC2335261

[7] Murad MH, Wang Z (2017) Guidelines for reporting meta-epidemiological methodology research. Evid Based Med 22(4):139–142 28701372 10.1136/ebmed-2017-110713 PMC5537553

[8] Kuitunen I, Räsänen K (2024) Non-invasive neurally adjusted ventilatory assist (NIV-NAVA) reduces extubation failures in preterm neonates-a systematic review and meta-analysis. Acta Paediatr 113(9):2003–2010. 10.1111/apa.17261 38703014 10.1111/apa.17261

[9] Zhu X, Qi H, Feng Z, Shi Y, De Luca D (2022) Noninvasive high-frequency oscillatory ventilation vs nasal continuous positive airway pressure vs nasal intermittent positive pressure ventilation as postextubation support for preterm neonates in China. JAMA Pediatr 176(6):551–559 35467744 10.1001/jamapediatrics.2022.0710 PMC9039831

[10] Wang K, Yue G, Gao S, Li F, Ju R (2024) Non-invasive high-frequency oscillatory ventilation (NHFOV) versus nasal continuous positive airway pressure (NCPAP) for preterm infants: a systematic review and meta-analysis. Arch Dis Child-Fetal Neonatal Edition 109(4):397–404 10.1136/archdischild-2023-325681 38228382

[11] Wang K, Zhou X, Gao S, Li F, Ju R (2023) Noninvasive high-frequency oscillatory ventilation versus nasal intermittent positive pressure ventilation for preterm infants as an extubation support: a systematic review and meta-analysis. Pediatr Pulmonol 58(3):704–711 36372443 10.1002/ppul.26244

[12] Kuitunen I, Räsänen K, Gualano MR, De Luca D (2024) Blinding assessments in neonatal ventilation meta-analyses: a systematic meta-epidemiological review. Neonatology 11:1–8. 10.1159/000539203 10.1159/000539203 PMC11633896 38861954

[13] De Luca D, Carnielli VP, Conti G, Piastra M (2010) Noninvasive high frequency oscillatory ventilation through nasal prongs: bench evaluation of efficacy and mechanics. Intensive Care Med 36(12):2094–2100 20857278 10.1007/s00134-010-2054-7

[14] De Luca D, Piastra M, Pietrini D, Conti G (2012) Effect of amplitude and inspiratory time in a bench model of non-invasive HFOV through nasal prongs. Pediatr Pulmonol 47(10):1012–1018 22328295 10.1002/ppul.22511

[15] Zhu X, Li F, Shi Y, Feng Z, De Luca D (2023) Effectiveness of nasal continuous positive airway pressure vs nasal intermittent positive pressure ventilation vs noninvasive high-frequency oscillatory ventilation as support after extubation of neonates born extremely preterm or with more severe respiratory failure. JAMA Netw Open 6(7):e2321644 37399009 10.1001/jamanetworkopen.2023.21644 PMC10318479

[16] De Luca D (2017) Noninvasive high-frequency ventilation and the errors from the past: designing simple trials neglecting complex respiratory physiology. J Perinatol 37(9):1065–1066 28904405 10.1038/jp.2017.84

[17] Loi B, Sartorius V, Vivalda L, Fardi A, Regiroli G, Dellacà R et al (2024) Global and regional heterogeneity of lung aeration in neonates with different respiratory disorders: a physiological observational study. Anesthesiology. 10.1097/ALN.0000000000005026 38657112 10.1097/ALN.0000000000005026

[18] De Luca D, Autilio C, Pezza L, Shankar-Aguilera S, Tingay DG, Carnielli VP (2021) Personalized medicine for the management of RDS in preterm neonates. Neonatology 118(2):127–138. 10.1159/000513783 33735866 10.1159/000513783

[19] Willhelm C, Girisch W, Gottschling S, Gräber S, Wahl H, Meyer S (2013) Systematic Cochrane reviews in neonatology: a critical appraisal. Pediatr Neonatol 54(4):261–266. 10.1016/j.pedneo.2013.03.002 23602385 10.1016/j.pedneo.2013.03.002

[20] Mandel D, Littner Y, Mimouni FB, Lubetzky R (2006) Conclusiveness of the Cochrane neonatal reviews: a systematic analysis. Acta Paediatr 95(10):1209–1212. 10.1080/08035250600580537 16982491 10.1080/08035250600580537

[21] De Luca D, Minucci A, Tripodi D, Piastra M, Pietrini D, Zuppi C et al (2011) Role of distinct phospholipases A2 and their modulators in meconium aspiration syndrome in human neonates. Intensive Care Med 37(7):1158–65 21567110 10.1007/s00134-011-2243-z

[22] De Luca D, Tingay DG, van Kaam A, Brunow de Carvalho W, Valverde E, Roehr CC et al (2016) Hypothermia and meconium aspiration syndrome: international multicenter retrospective cohort study. Am J Respir Crit Care Med 194(3):381–4 27479063 10.1164/rccm.201602-0422LE

[23] Aguayo-Albasini JL, Flores-Pastor B, Soria-Aledo V (2014) GRADE system: classification of quality of evidence and strength of recommendation. Cir Esp 92(2):82–88 24361098 10.1016/j.ciresp.2013.08.002

[24] Gao Y, Yang K, Cai Y, Shi S, Liu M, Zhang J et al (2020) Updating systematic reviews can improve the precision of outcomes: a comparative study. J Clin Epidemiol 125:108–19 32442481 10.1016/j.jclinepi.2020.05.019

[25] Djulbegovic B, Ahmed MM, Hozo I, Koletsi D, Hemkens L, Price A et al (2022) High quality (certainty) evidence changes less often than low-quality evidence, but the magnitude of effect size does not systematically differ between studies with low versus high-quality evidence. J Eval Clin Prac 28(3):353–62 10.1111/jep.13657 PMC9305903 35089627

